# New Rocaglate Derivatives Tip the Scale against Brain Tumors

**DOI:** 10.1021/acscentsci.4c01524

**Published:** 2024-10-08

**Authors:** Shintaro Iwasaki

**Affiliations:** † RNA Systems Biochemistry Laboratory, RIKEN Cluster for Pioneering Research, Wako, Saitama 351-0198, Japan; ‡ Department of Computational Biology and Medical Sciences, Graduate School of Frontier Sciences, The University of Tokyo, Kashiwa, Chiba 277-8561, Japan

Targeting cellular translation pharmacologically offers a promising approach for cancer therapy, as dysregulated protein synthesis is closely linked to tumorigenesis. Due to their strong antitumor effects, translation inhibitors termed rocaglates have attracted attention.^1^ Despite extensive efforts to survey effective derivatives,^1−4^ the tailoring of rocaglates for specific tumor types remains incomplete. In a recent issue of *ACS Central Science*, Sunil K. Malonia, John A. Porco, Jr., and co-workers developed unique rocaglate derivatives, termed rocaglate acyl sulfamides (Roc ASFs), that effectively and selectively suppress glioblastoma stem cells, discriminating them from nonstem cancer cells.^5^

**Figure 1:**
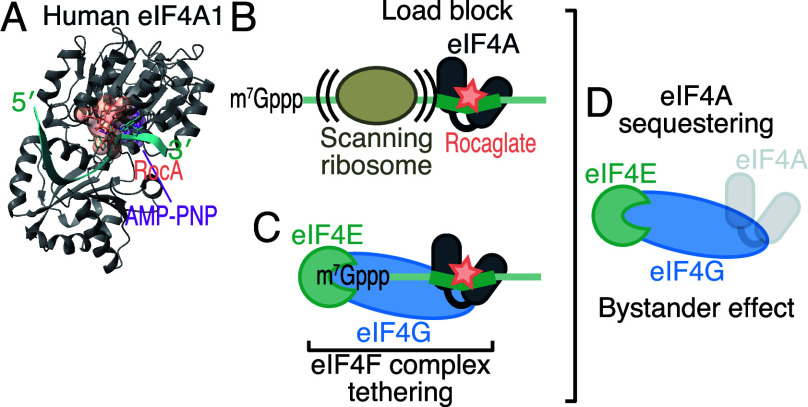
(A–D) Schematic of the mode of actions of rocaglates in translation repression. For A, the structure of human eIF4A1·rocaglamide A·polypurine RNA·AMP-PNP complex (5ZC9) is shown.

**Figure 2:**
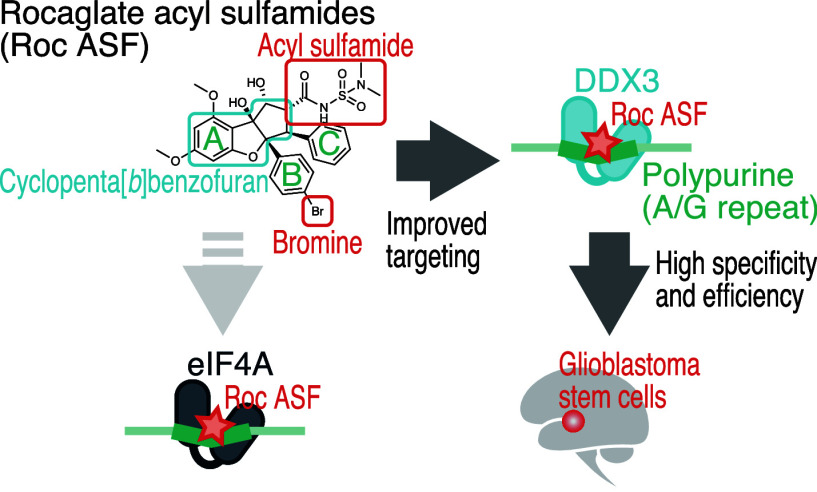
Schematic of the chemical structure of Roc ASF and the improved affinity toward DDX3 for the specific cytotoxicity for glioblastoma stem cells.

Rocaglates are small molecules, which were first identified as natural products from *Aglaia* trees. Following the discovery of antitumor effects, significant efforts have been made to investigate the underlying molecular events induced by the compounds. Primarily, rocaglates repress protein synthesis. This effect is attributed to targeting DEAD-box RNA binding proteins eIF4A and DDX3^6,7^—factors pivotal for translation initiation. Rocaglates do not induce simple loss-of-function of these proteins; rather, they provide their gain-of-function effects and exert a distinctive and complex influence on protein synthesis. Although eIF4A and DDX3 do not directly associate with RNA bases, rocaglates convert these proteins to A/G repeat (i.e., polypurine)-selective RNA binding proteins^6−8^ (Figure 1A). This new RNA sequence selectivity is mediated by direct base recognition by a rocaglate fitted into the interfacial pocket between eIF4A/DDX3 and polypurine RNA.^6^ The biochemical conversion of the target DEAD-box RNA binding proteins induces adverse effects on translation: 1) serving eIF4A/DDX3 on the polypurine motif as a load block for scanning ribosomes^7,8^ (Figure 1B), 2) tethering eIF4F—a trimeric complex of eIF4A, eIF4E, and eIF4G, on the 5′ cap structure when polypurine is proximal to it^3^ (Figure 1C), and 3) sequestering eIF4A/DDX3 on polypurine RNA from a new round of translation of other mRNAs (i.e., bystander effect)^3^ (Figure 1D). Due to the dominant negative effects,^7^ higher expression of eIF4A/DDX3 in a subset of tumor cells may be a determinant for the cytotoxicity. Also, cancer cells associated with aneuploidy^1^ and those driven by MYC activation^9^ are known to be sensitive to rocaglates. The development of rocaglate derivatives that sharply distinguish tumor cells from nontumor cells has been a demanding task.

To tackle this issue, the authors systematically synthesized rocaglate derivatives, which have a cyclopenta[*b*]benzofuran core (Figure 2) and investigated their effects on cell viability in glioblastoma stem cells and nonstem cancer cells.^5^ Generally, rocaglates demonstrated the potential to eliminate glioblastoma stem cells selectively. Through structure–activity relationships (SARs), the authors found that C4*′*-bromination at the B-ring and C2-acyl sulfamoylation (Figure 2) increased the potency of the rocaglates against cancer stem cells. The authors termed this subgroup of rocagaltes as Roc ASF (Figure 2).

Harnessing a derivative of the mass spectrometry-based cellular thermal shift assay (Proteome Integral Solubility Alteration assay or PISA assay), the authors demonstrated that Roc ASF preferentially targets DDX3 over eIF4A. This was further supported biochemically, as Roc ASF showed an improved affinity for DDX3. *In silico* modeling of Roc ASF binding to DDX3 suggested that bromine at C4′ and acyl sulfamide at C2 may improve engagement with the protein.

Importantly, the anticancer stem cell activity of Roc ASF was not simply associated with its translation inhibition. Compared to the other types of rocaglates, Roc ASF did not show better translation repression by the *in vitro* translation system. These data suggested that improved targeting to DDX3, rather than boosted translation repression, is key to cancer stem cell toxicity. Indeed, DDX3 is overexpressed in various cancer stem cells.^10^

This exciting development of Roc ASF simultaneously raises a series of new questions. Does Roc ASF repress translation in both glioblastoma stem cells and nonstem cells in a similar manner? If the translational repression *per se* is not a primary reason for cancer stem cell specificity, what kind of function was provided to DDX3 by Roc ASF? Since DDX3 has multifaceted roles in RNA metabolism other than translation,^10^ diverse malfunctions in RNAs could be speculated. Considering that DDX3 knockdown or loss-of-function-type pharmacological inhibition may also phenocopy Roc ASF,^5^ simple sequestration of DDX3 or the bystander effect could be the main cause. DDX3 mutations also cause glioma.^10^ How could the suppression of the DDX3’s function by RocA ASF and the tumorigenetic DDX3 mutations be reconciled? Optimizing rocaglates to target DDX3 and detailed investigations in RNA metabolism may open new avenues for drug development toward brain tumors.
